# Harnessing opportunities for good governance of health impacts of mining projects in Mongolia: results of a global partnership

**DOI:** 10.1186/s12992-017-0261-5

**Published:** 2017-06-27

**Authors:** Michaela Pfeiffer, Delgermaa Vanya, Colleen Davison, Oyunaa Lkhagvasuren, Lesley Johnston, Craig R. Janes

**Affiliations:** 1 Interventions for Healthy Environments, Department of Public Health, Environmental and Social Determinants of Health, World Health Organization, Av Appia 20, CH-1211 Geneva 27, Switzerland; 2 Environmental Health, WHO Country Office, Government building VIII, Olympic Street- 2, Sukhbaatar District, Ulaanbaatar, Mongolia; 3 Department of Public Health Sciences, Queens University, Ontario, Kingston Canada; 4 Leading Researchers, NGO, Ulaanbaatar, Mongolia; 5 School of Public Health and Health Systems, University of Waterloo, Waterloo, ON N2L3G1 Canada; 6 Simon Fraser University, Burnaby, BC V3L1S7 Canada

**Keywords:** Sustainable development goals, Mining, Mongolia, Environmental impact assessment, Health impact assessment, Intersectoral partnerships, Policy change, Capacity development

## Abstract

**Background:**

The Sustainable Development Goals call for the effective governance of shared natural resources in ways that support inclusive growth, safeguard the integrity of the natural and physical environment, and promote health and well-being for all. For large-scale resource extraction projects -- e.g. in the mining sector -- environmental regulations and in particular environmental impact assessments (EIA) provide an important but insufficiently developed avenue to ensure that wider sustainable development issues, such as health, have been considered prior to the permitting of projects.

**Methods:**

In recognition of the opportunity provided in EIA to influence the extent to which health issues would be addressed in the design and delivery of mining projects, an international and intersectoral partnership, with the support of WHO and public funds from Canadian sources, engaged over a period of six years in a series of capacity development activities and knowledge translation/dissemination events aimed at influencing policy change in the extractives sector so as to include consideration of human health impacts.

**Results:**

Early efforts significantly increased awareness of the need to include health considerations in EIAs. Coupling effective knowledge translation about health in EIA with the development of networks that fostered good intersectoral partnerships, this awareness supported the development and implementation of key pieces of legislation. These results show that intersectoral collaboration is essential, and must be supported by an effective conceptual understanding about which methods and models of impact assessment, particularly for health, lend themselves to integration within EIA.

**Conclusions:**

The results of our partnership demonstrate that when specific conditions are met, integrating health into the EIA system represents a promising avenue to ensure that mining activities contribute to wider sustainable development goals and objectives.

## Background

The Sustainable Development Goals call for the effective governance of shared natural resources in ways that support inclusive growth, safeguard the integrity of the natural and physical environment, and promote health and well-being for all [1]. For large-scale resource extraction projects such as those in the mining sector, environmental regulations that require impact assessments prior to project permitting, as well as appraisals of cumulative regional effects, offer a critically important entry point to ensure that wider sustainable development issues, including health, are considered in governance of the resource sector. A particularly important entry point is the environmental impact assessment (EIA) [2]. Applied nearly universally around the world, the EIA system offers the potential to link multiple public and private sector actors in the provision of important public health goods.

The extent to which EIA processes are capable of marshaling multiple sectors to reduce risks to health, wellbeing, and health equity at the level of the community is neither well understood nor researched. Although national regulations of impact assessments commonly include requirements related to the assessment of human health impacts, in practice, inclusion of health in EIAs is often incomplete and limited to coverage of physical environmental considerations (such as air, water, soil and pollution/emissions-related issues). Other important factors with implications for health, for example related to health determinants affected by changes to the social environment (e.g., population influx, impacts on public health infrastructure, or the social determinants of health more generally), are not often included or are considered separately, for example as part of other types of assessments that may or may not be considered in project appraisal [2, 3]. The resulting picture of health that emerges from EIAs is often incomplete [4, 5]. This is not only a major missed opportunity, but may contribute – however unwittingly – to the development of unanticipated but significant adverse health effects.

Health risks that are not adequately identified early in the EIA process can pose unnecessary costs to projects and in some instances may threaten the achievement of the project itself. At the same time, opportunities for projects to positively contribute to the health and well-being of surrounding communities can go unnoticed or be underleveraged if health issues are not fully considered as part of project due diligence and planning. Assessments that consider impacts across the broad determinants of health have been identified as a promising avenue to leverage public investments in the social sector [6]. The Health Impact Assessment (HIA), which grew out of efforts to integrate equity and social determinants of health perspectives into the assessment process, represents a particularly significant effort to do this [7]. HIA methods, tools, and processes have developed over the past few decades, but have mainly been applied in high income countries, in particular Europe and Australia [7]. Attempts to adapt HIAs to projects in low and middle-income country (LMIC) settings have exposed many limitations to the approach, in particular the limited capacity of governments to manage impact assessment processes [8]. Yet important lessons have been learned and applied in both public and private sectors, and the HIA is now championed globally as a cornerstone of sustainable development [6, 9]. However, it remains to be seen whether HIA systems can be fully established in LMIC contexts. A more promising, and perhaps transitional approach, is to bring HIA perspectives, tools and methods into existing and functioning EIA systems. Integrating considerations of social, cultural, and health resources into the EIA process provides a particularly tractable approach to effective environmental and health governance, especially in settings where extractive industry development is dominant [2].

Mongolia is a particularly important site for advancing such integration. The extractives sector accounts for over 87% of exports, 20% of GDP, and nearly one-third of the state budget. In 2015, the value of mineral exports as a proportion of all exports were: copper concentrate 48%, coal 13%, crude oil 8%, iron ore 6%, gold 13%, zinc 3%, and others 1%. Cashmere (1% of total export value in 2015) is the major non-mineral export commodity [10]*.* Coal exports dropped dramatically from nearly one-half of total export value in 2012 to just 13% in 2015. Increasing exports of copper concentrate from the major Mongolia-Russian joint Erdenet Mining Corporation and the scale-up of mining operations at the Rio Tinto-Mongolian operation at Oyu Tolgoi in the southern Gobi has made this mineral an increasingly valuable export commodity [10].

Driven largely by the rapidly expanding mining sector, from 2010 to 2013 the country experienced strong double-digit economic growth. However, in 2015 the weakening of the global commodity market for copper and coal, coupled with a faltering Chinese economy, Mongolia’s main export partner, slowed GDP growth to 2.3% in 2015 [11]. At this writing (October 2016), exports, foreign investment, and GNI per capita have all continued to decline and public debt has soared to an alarming level [12].

Mongolia’s current population is just over 3.0 million, with two-thirds living in urban areas [13]. The poverty rate declined steadily over the past decade, dropping from 39% in 2010 to just below 22% in 2015, though many remain near the poverty line [12]. Although GDP per capita has increased over the last ten years and was USD$ 4280 in 2015, economic inequality has increased [11, 14]. These economic difficulties notwithstanding, and given the development potential associated with this abundant mineral resource wealth, mining and related industrial developments -- e.g. in energy, road and rail infrastructure, ore processing and transportation -- are considered central to the continued growth and development of Mongolian society.

Many of these developments can have positive and beneficial impacts for the population as a whole, for example where new income and employment opportunities are created and where there are substantial improvements in basic infrastructure and social services. Some impacts, however, may not be so positive, particularly for communities near mining and ancillary sites of operation. This is already becoming evident in some parts of the country, such in the southern Gobi region where there are several large coal and copper mining operations and where the rapid influx of workers and job seekers has changed local population dynamics and placed considerable stresses on social and health services. For example, the National Statistical Office of Mongolia reports that the population of two districts in the Gobi where mining is dominant have increased as much as five-fold since 1989 [13]. Work done by Janes and colleagues in mining affected areas of the south Gobi identified likely project risks associated with population influx, including a lag in infrastructure development, overcrowded and unsanitary living conditions, increased use of alcohol with attendant psychosocial risks, increases in commercial and unsafe sex, disruption to families, increased injury rates related to motor vehicle accidents, and inadequate infrastructure for food transport and storage [15–17]*.*

For these reasons, and motivated by specific concerns over the impacts of rapid extractive industry development on the health and well-being of Mongolian communities, Mongolian and Canadian academic and public health practitioners came together to find ways to foster better inclusion of human health, broadly defined, in the environmental impact assessment process. Through these efforts, we established a multi-institutional and -country partnership through which we worked to enhance coverage of health in impact assessments conducted in Mongolia’s extractives sector.^1^ Over a period of six years this international partnership undertook several discrete but linked knowledge mobilization and advocacy projects, focused principally on effecting changes to EIA governance processes.

The objective of this partnership has been to effect policy changes across relevant public institutions to ensure adequate coverage of health issues in the review and permitting process, influencing the extent to which health issues would be addressed in the design and delivery of mining projects, thereby improving the governance of the extractives sector. Our intervention strategy was based on principles of integrated knowledge translation, which is an approach that engages potential knowledge-users as partners in the research process [18]. Following this approach, the different activities we discuss here were not part of a single policy intervention scripted at the outset, but consisted of linked and mutually supportive and synergistic projects, culminating in intensive capacity building activities designed to catalyse intersectoral partnerships and solidify capacity for including human health in an evolving EIA process. The interventions were iterative, each building on the outcomes and opportunities created by the previous one.

## Methods

Six intersectoral knowledge mobilization interventions were implemented between 2009 and 2015. These interventions are summarized in Table 1. The initial impetus for a focus on mining and health in Mongolia grew out of a 2007 consultation on health research priorities organized by Canadian and Mongolian members of the Canadian Coalition for Global Health Research [19]. Our first effort was to organize an international workshop in 2009 to bring together Mongolian and international researchers to discuss the impacts – both documented and potential – on mining and public health in the Mongolian context. At the conclusion of this workshop participants agreed to an action agenda that called for assembling, synthesizing, and mobilizing knowledge on the public health impacts of extractive industry development, with a specific focus on the Mongolian setting. This action agenda provided both the impetus and framework for the projects that followed.

**Table 1:** Interventions aimed at facilitating better coverage of health in EIA practice in the Mongolian Extractives Sector, 2009–2015

Intervention type	Main stakeholder addressed/target audiences	Scope of focus/ thematic orientation	Objectives	Activities [references]	Key Outcomes	Dates
*International Conference* Awareness raising / sensitization	Canadian and Mongolian researchers, representatives of the mining industry, public health scientists, and Mongolian policymakers	Health impacts of mining activities	To assess the current state of knowledge about mining impacts in Mongolia, identify research and policy gaps, and to develop a strategy for addressing these gaps.	International conference in Ulaanbaatar [19]	Established framework for moving forward with a mining and health knowledge translation/mobilization agenda.	2009
*KT-1* Technical support to develop specific tools and methods	Canadian and Mongolian researchers, representatives of the mining industry, public health scientists, and government policymakers	Addressing issues of health equity in the extractives sector.	To develop consensus on a methodology and tools for implementing a social determinants of health and equity-focused health impact assessment that would be relevant to Mongolian communities. To provide evidence-based support for and encourage ongoing efforts to strengthen the regulatory environment surrounding impact assessments.	Organized and met with intersectoral working group; held two day intensive retreat focusing on health equity within HIAs; project team visited communites to scope health impacts of mining [20, 22]	Awareness of utility of equity-focused HIAs in the extractives sector; development of introductory equity-in-HIA tool; targeted technical support for the development of tool and supporting materials; training and capacity building activities to support the use of the tool; awareness of need to address health consequences of mining among key stakeholders.	2010
*WHO-1* Awareness raising/sensiti-zation	Representatives of multiple government ministries and academic institutions	Introduction to health impact assessment (HIA)	To raise overall awareness, in particular among health actors, of basic concepts and methods used in HIA.	Stakeholder consultations (to inform development of training materials); conduct of a training workshop on HIA methods [5]	Increased awareness of HIA concepts and methods.	2011
*KT-2* Training/ capacity building on use of specific HIA tools, development of a mining and health strategy within the Mongolia Ministry of Health	Canadian and Mongolian researchers, representatives of the mining industry, public health scientists, and government policymakers	Health equity impact assessment tools and methods; health impact assessment as a key component of a mining and health strategy.	To continue to build capacity to support the use of equity-focused HIA methods in the extractives sector.	Continued meeting with intersectoral working group from KT-1 project; targeted technical support for the development of HIA methods and processes; continued awareness raising among key stakeholders [15, 20]	Expanded awareness of HIA as a key element of a mining and health strategy; support for changes to the EIA law; established cross-ministry working group on HIA.	2011–12
*WHO-2* Awareness raising /sensitization, training/capacity building	Representatives from the Ministry of Environment (at both central and provincial levels) responsible for regulating the EIA process, and their MOH counterparts.	Health in the environmental impact assessment process	To sensitize EIA regulators and their health sector counterparts about where (as in at what points in the process) health issues could be better addressed in Mongolia’s EIA process.	Training workshop and provision of targeted technical support to develop guidance how to include health at different points in Mongolia’s EIA system [2]	Increased capacity to apply health impact assessment methods within the EIA process; support for new EIA law.	2014
*HIA-LDP* Training/ capacity building on use of specific tools and methods	Representatives of multiple government offices responsible for health, environment,mining, justice, food & agriculture; local officials, academics from the Medical University, civil society organizations, international participants from South Korea, Canada, Zambia, and Tanzania	Tools and methods to address health in EIA	To build capacity of key EIA actors across sectors to address health issues associated with extractive industries activities.	8-day intensive learning program focused on building institutional capacity to review, manage, and regulate health in EIA processes [21]	Increased institutional capacity to apply health impact assessment methods within the EIA process; developed networks of HIA “experts” in different institutions; support of the new hygiene law.	2015

The team received funding for two knowledge translation and dissemination projects, conducted between 2010 and 2012, that focused on identifying international best practices for researching and addressing the health impacts of extractive industry development (see Table 1). The objectives of the first project (KT-1), begun in 2010, were to: further develop an international partnership tasked with addressing health inequities associated with the growth of Mongolia’s mining sector; to develop consensus on a methodology and tools for implementing a social determinants of health and equity-focused health impact assessment (HIA) that would be relevant to Mongolian communities; and to provide evidence-based support for and encourage on-going efforts to strengthen the regulatory environment surrounding impact assessments. The second KT project (KT-2), initiated in 2011, continued these knowledge mobilization efforts with goals of disseminating newly developed tools, expanding partnerships and increasing outreach efforts to larger numbers of stakeholders. Both projects involved three to four working group consultations over a period of one year each, with each culminating in multiday health impact assessment and mining and health strategy workshops: one in Ulaanbaatar, Mongolia; and one in Vancouver, Canada.

It is important to note here that early awareness raising and knowledge mobilization projects included large numbers of representatives of affected public and private sectors. Stakeholders involved at various points included senior-level staffs from the Ministry of Health (MOH), the Ministry of Environment (MOE), the Ministry of Mining (MOM), the State Inspection Agency, the Medical Sciences University of Mongolia, intergovernmental development organizations (United Nations Population Fund (UNFPA), United Nations Children’s Fund (UNICEF), the United Nations International Labour Organization (UN-ILO), and the World Health Organization (WHO)), and several civil society organizations.^2^ Members of affected communities were invited to participate in the health impact assessment workshops. Representatives of the community relations offices of two major mining companies, Energy Resources, LLC (a Mongolia-owned and operated coal mining company), and Turquoise Hill (a copper-mining operation owned jointly by the Government of Mongolia and Rio Tinto) were active participants in these activities.

In 2011, WHO-Mongolia organized a separate consultancy on HIA, which included a well-attended workshop on HIA concepts and methods (WHO-1). This workshop did not focus specifically on the extractive sector, but on assessing development impacts more broadly [5]. To build on the positive results achieved through earlier efforts, in late 2014 and mid-2015 we organized two intensive learning activities designed to develop capacity to implement health assessments within a newly developed Mongolia environmental impact assessment process (described further below). The first, organized by WHO, was a three-day workshop with a custom, purpose-built curriculum (WHO-2). The second, initiated by the Canadian Coalition for Global Health Research (CCGHR), was a two-week intensive institute, the “Health Impact Assessment Learning Development Program” (HIA-LDP). Both the WHO and CCGHR initiated activities focused training efforts on policymakers and change agents across relevant sectors.

WHO-2 was organized in Ulaanbaatar in November, 2014, and focused on raising awareness about where and how health issues could be better addressed in the EIA process, for example during screening, scoping or appraisal review. New WHO training materials on health in EIA were designed and used for this purpose. The workshop was conducted in Ulaanbaatar and included EIA officers from central Ministries responsible for health and EIA, as well as EIA and health officers from ten of the provinces in Mongolia that are host to significant mining operations. Representatives of the government inspection agency responsible for monitoring and verification of compliance on environmental permits, and their provincial counterparts from ten mining affected provinces, were also present.

The HIA-LDP, which was held in April and May 2015 in the southern Gobi provincial capital of Dalanzadgad, with a final wrap-up meeting in Ulaanbaatar, sought to build on and extend the awareness raised in the preceding WHO workshop (WHO-2) to develop capacity for the use of specific methods and tools to support the assessment of health in EIA, primarily during the analytical phase. This intensive two-week training was designed to build capacity for including health in impact assessment processes. Participants included Mongolia government officials responsible for health, environment, mining, justice, food and agriculture, and inspection; local officials from southern Gobi province, academics from the School of Public Health at the Medical University, and representatives of civil society. It also included a group of international participants from South Korea, Tanzania, Zambia, and Canada interested in applying HIA concepts and methods to extractive sectors in their own countries. The curriculum was designed to further build the capacity of decision-makers and institutions involved in impact assessment regulation and training, to introduce principles of change leadership, and to culminate with groups presenting “action plans” for implementation in their respective organizations.

We evaluated the impact of these events on the policy environment in Mongolia in three formal ways: a 2012 qualitative assessment among key stakeholders of knowledge of health impact assessments and their applicability to the resource sector [15, 20]; an evaluation of legal innovations and policy changes (described below and updated in October, 2016); and pre- and post-activity survey and six-month follow-up of participants from the final (2015) intensive HIA-LDP [21]. Research ethics approvals for survey and interview elements of the evaluation were obtained from Simon Fraser University; the University of Waterloo; and the Mongolia Ministry of Health.

## Results

### Early stage awareness-raising – Creating buy-in and building partnerships, 2009–2012

Having identified health impact assessment (HIA) as a promising policy intervention to mitigate the health impacts of mining, our project team’s^1^ early efforts at policy change focused on engaging multiple stakeholders in discussions regarding the applicability of HIA concepts and methods to the Mongolian context, and encouraged influential policymakers to consider whether HIA should and could form the cornerstone of an overarching mining and health strategy [15, 20, 22]. These multi-stakeholder engagement activities focused principally on workshopping best practices for assessing health impacts in the resource sector, including application of concepts of health equity within health impact assessments, and tailoring and applying impact assessment processes to the Mongolian context. The rationale behind this approach was to try to create interest for better coverage of health in Mongolia’s EIA system and to sow seeds that would later support the intersectoral collaboration needed to deliver health-inclusive EIAs.

In 2012, we paused to assess our progress to that point in mobilizing knowledge about HIA within the extractives sector. Key achievements included development of a simplified equity-based HIA tool appropriate to the Mongolian context. The document was translated into Mongolian and distributed widely to private and public sector stakeholders [20, 22]. Dissemination of the tool was accompanied by a series of workshops and meetings in Ulaanbaatar, as well as in communities in the south Gobi that had been affected by mining development. Interviews with stakeholders across public, academic, and private sectors indicated that knowledge of the diverse and multi-sectoral health impacts of mining activities had been diffused across policy domains [15, 20]. Importantly, there were clear signs of cooperation between the formerly siloed Ministries of Health and Environment, and an appreciation of the importance of applying a broader determinants of health approach within the EIA process. Although it is difficult to assess the specific causal relationship between our KT-1, KT-2, and WHO-1 projects and subsequent policy changes, it is certainly the case that diffusion of knowledge of HIA and applications of HIA in EIA among key policy networks provided support for the development and implementation of two highly significant changes in the public, regulatory sector: revision to the Law on Environmental Impact Assessment and reformulation of a Law on Hygiene [20].

### Strengthening the legal environment, 2012–2015

#### The revised law on environmental assessment

With support from our partners in the Ministry of Health, in May 2012 the Government of Mongolia revised the legal framework governing the environmental impact assessment process. The original environmental laws, passed in 1998 and amended once in 2001, were considered by many to be inadequate to govern the rapid development of the extractives sector. The 2012 Law on EIA was written to include more specific and detailed provisions and requirements for the consideration of health issues than the previous environmental regulations [23], and includes clear reference to the importance of HIAs. For example, article 7.7 of the EIA law states that impact assessments must include “social and health impact assessments.” The new law also consolidated and streamlined several related processes and procedures, for example related to cumulative environmental impact assessment, public participation, and stakeholder and community engagement. Subsequent to the passage of the EIA Law, recommendations were developed in 2013 that stipulated how EIAs were to be conducted, including specific steps and requirements. These were formalized when specific health requirements were included as an annex to the formal EIA regulations that were approved by Government decree on the 16th of November 2013.

At about this time, efforts were begun by officials in the health sector, including members of our partnership team, to integrate HIA into development of a new public health law – the Law on Hygiene (see below). This new law, formalized finally in 2016, informed requirements related to the assessment of health issues within the EIA process, though further work is needed to clarify how the two pieces of legislation will coincide.

#### The law on hygiene

Reflecting the separate, but synergistic, interests of the relevant Ministries – the Ministry of Environment wanting to take on the issue of EIA regulations and the Ministry of Health wanting a more specific focus on HIA regulations – initial policy focus within the Ministry of Health was directed toward the introduction of a new national public health law on hygiene. This law, based loosely on the provisions of a 1998 “Law on Sanitation,” was envisaged to constitute a legal basis for requiring that health impact assessments be conducted on any major development initiative, including mining projects. Despite its origins within the health sector, and largely as a result of the different project initiatives described here, there were efforts by cross-Ministry working groups to ensure some consistency between EIA and HIA regulations. Approved by the Mongolian Parliament in February 2016, the Law on Hygiene has a separate article that focuses on health impact assessment that parallels related provisions in the Law on EIA for conducting health assessments within EIAs applied to new development projects. Also included in the law are provisions related to the conduct of independent or stand-alone health assessments for some projects that might influence or could have influenced human health negatively.

Although recognized as a significant success from a public health perspective, and despite efforts to align regulations, the relationship between the newer hygiene law and the revised and amended EIA law as yet remains unclear. The EIA law specifies the importance of HIA in assessing cumulative impacts of projects. It also specifies that assessment of human health impacts is a cornerstone of EIA methodologies. Conversely, it is uncertain whether the law on hygiene goes beyond occupational health and safety issues related to the working environment to assess wider environmental and social factors linked to projects at the proposal stage. The hygiene law as written appears to come into effect during construction and operation, which means that if health issues were identified at this stage (i.e. after a project was approved and after the environmental permit was issued) it would be difficult to make substantive changes to the overall design of a project. The amended EIA law, however, comes into effect earlier in the project planning and decision-making process (i.e. prior to project approval and the issuance of an environmental permit). Because of this, and because the changes to the EIA law provide a stronger legislative basis for health assessment, public health stakeholders have given priority to enhancing coverage of health within the existing EIA system as a means of enhancing governance of social and, in particular, health risks associated with the rapid development of Mongolia’s mining economy.

Although the new Law on Hygiene may have limited relevance for governance of mining projects prior to permitting, it is important to note that the process of developing the law has significantly enhanced the profile of HIAs in governance of the extractives sector. For example, in 2014 an intersectoral thematic working group on HIA was established by a joint order of the Ministries of Health and Environment. This working group -- which includes representatives from government, academic research institutions, civil society, and international development organizations -- is intended to provide technical support for implementing all activities within the EIA law, including conducting assessments, mitigating health impacts, and strengthening human resource capacity. Shortly after the thematic working group was established, the Mongolia Public Health Institute, a research and practice unit associated with both the Mongolia Academy of Science and the Ministry of Health, established a task force to support the development of the EIA and Hygiene laws, develop regulatory documents, and provide guidance to conducting HIAs.

While these efforts contributed to a clear operational framework for addressing health in EIA in Mongolia, specific roles, responsibilities and inputs of different actors involved in the EIA process, in particular health actors, needs to be further clarified. Institutional capacity to support the operationalization of health in EIA also needed to be strengthened at both the national and sub-national levels. Further guidance related to practice standards for health impact assessments is also under development and will form part of the suite of regulatory materials influencing HIA practice in Mongolia. The final two interventions we describe here were developed specifically to meet these needs (see Table 1).

### Building capacity and creating an enabling environment: Results of the 2014 and 2015 training programmes

To build institutional capacity for HIA within EIA, the 2014 WHO workshop (WHO-2) sought to raise awareness about where and how health issues could be better addressed in the EIA process, for example during screening, scoping or appraisal review. The WHO worked with a group of international experts to develop training materials for this purpose. The second activity, the CCGHR-led HIA-LDP, sought to build on and extend this awareness to develop capacity for the use of specific methods and tools to support the assessment of health in EIA. Both activities focused principally on governance issues; neither was designed to train impact assessment practitioners in Mongolia, although it was anticipated that some participants might take this on as a subsequent activity within their respective organizations. The target audiences for the two activities were similar, though there was only a slight overlap of attendees, and included EIA regulatory actors and their health sector counterparts, i.e. those responsible for ensuring the implementation of the EIA Law and related regulations, as well as representatives from Mongolia academic, research, and civil society organizations.

A formal evaluation of the CCGHR HIA-LDP was completed initially post-activity, and following a period of six months [21]. Analysis of the results show that relationships formed during the training were maintained six-months later, with participants reporting that they continued to support one another in projects related to health impact assessments either independently, or within the EIA context. Relationships were formed across Ministries, which further supported and catalyzed intersectoral partnerships established by the projects and activities described here. Several participants noted that their participation in the training had conferred on them the label of “HIA expert” within their organizations, with some reporting that they were newly engaged in health systems change, re-writing laws and amending regulatory processes, including those related to the Law on Hygiene discussed above, and creating guidelines to further strengthen HIA and EIA policy. Others were newly energized to develop curricula within universities and training institutes to train impact assessment practitioners. Although the participants reported significant progress, they also recognized the need for continued and broad recognition of health impacts in the mining sector, and that more education and further training across ministries were clearly needed [21]. In addition, many were concerned with the political instability that plagues Mongolia – with each election cycle there is often wholesale turnover of senior civil service professionals – and an increasingly precarious economy which tends to push health and social welfare to the sidelines in order to boost extractive sector development and production.

A notable element of our knowledge translation and mobilization efforts over the past six years was a productive, and to some degree synergistic, engagement with the private sector. Community relations staff from two major mining concerns – the Turquoise Hill/Government of Mongolia copper mine at Oyu Tolgoi, and the Energy Resources’ coal operation at Tavan Tolgoi,^3^ − participated actively in early workshops. In 2010, the operators of Oyu Tolgoi, as required by loan covenants, implemented a “Community Health, Safety, and Security Program,” which included implementation of an independent HIA.^4^ Members of the consortium who won the contract to conduct the HIA participated in our KT-1 workshops and contributed to the development of the Mongolia HIA-Equity tool [20, 22]. One of our core project team provided external, arms-length consultation on the HIA process and evaluated the final product, risk matrix, mitigation plan, and budget. During the KT-1 process, several members of our partnership also visited both companies and nearby affected communities in order to observe community relations and health efforts first hand. Information gained from these visits informed the development of the HIA-LDP curriculum implemented in 2015. Input from our team into the Oyu Tolgoi HIA and mitigation plans also informed the development of specific program elements.

## Discussion

The focus of our dissemination, knowledge translation, and advocacy efforts changed over time. Early efforts focused on introducing the concept of health impact assessment, health equity and varied determinants of health within the assessment process, and analyzing the relevance of health impact assessment to strategic planning in the extractives sector within the Ministry of Health. These initial activities were important for establishing a basic level of knowledge about implementing, assessing and regulating impact assessment, and were successful in stimulating the diffusion of HIA as a governance strategy both within and outside of the health sector [20]. The passage of the new EIA law in 2012 led us to focus in a more concentrated way on building capacity amongst key actors in the EIA system. The 2014 and 2015 efforts, which focused on providing support to regulators at both local and national levels, policy makers, academics, and civil society, were unique. Building on early policy wins, these later efforts broadened understandings of the importance of including community perspectives and community members in the EIA process, increasingly addressed how HIA relates to EIA, and how to build HIA into Mongolia’s highly regulated EIA system.

Due to vast differences in conceptual understandings about impact assessments (EIA, HIA or other), and often vague understandings about the determinants of health outside of the narrow domains of occupational or physical environmental health, significant time and effort in many of our activities was needed to establish a common language on key concepts. During this process important differences in understanding about the purpose and application of impact assessments were identified. For example, many of our early efforts to promote health impact assessment in Mongolia were based on models of HIA used to support “Health in All Policies” and action on the social determinants of health. This has very different origins (and objectives) than the approach to EIA applied in Mongolia, which focuses far more narrowly on air, water, and soil impacts. This remains, in our view, a significant obstacle in implementing HIAs within the EIA process. A lesson of our work is that more focused, early discussion of the determinants of health across domains would have provided a foundational language both for addressing the need for HIAs and for considering the breadth of mitigations across sectors – a likely outcome of such an approach.

For Ministry of Health staff, understanding these differences in perspective was important, and would eventually shape their overall perspectives on HIA. Although the EIA Law provided a strong legal framework for health impact assessment it would only facilitate its application at the project level and only in certain development project decision-making contexts, i.e. where EIAs were required. It would not facilitate or allow for other important applications of HIA, for example at the program and policy (e.g. sector-wide) levels. This meant that other channels, for example as provided in the hygiene law, still need to be developed in parallel to any efforts focused on health in EIA. It also means that while EIA may provide an important opening for managing health risks and benefits associated with mining and other complex development projects, it will not necessarily be a useful channel for addressing broader health objectives related to the institutionalization of HIA and Health in All Policies initiatives.

Both the WHO-led workshop in 2014 (WHO-2) and the CCGHR-led 2015 HIA-LDP focused mainly on enhancing capacity to address health within the existing EIA process, and particularly in the extractives sector. In this sense, both were successful in continuing to build a somewhat more narrow approach to HIA, and in particular to ensure that officials from different Ministries learned to use consistent language about HIA and the determinants of health [21]. Participants considered the workshops to be of direct relevance to their professional positions and responsibilities, and found that the action plans, presented publicly at the end of the HIA-LDP workshop, gave them a valuable opportunity to tailor elements of the standard HIA process to their roles within their respective organizations. Some of the participants from the Ministry of Health cited the latter workshop as supporting their involvement in the development and implementation of the 2016 Law on Hygiene. The academic participants used their new knowledge to develop HIA courses for public health students in the Medical University. Participants also valued the opportunities for relationship building across ministries and the shared capacity building offered through the program [21].

Inclusion of representative from the private sector, particularly in the KT-1 project, were noteworthy, both in informing the content of later activities, as well as supporting industry efforts to implement and act on HIAs. While it is impossible to assess the full impact of their participation, it is clear that interaction between our project team and these important corporate actors was significant. These interactions facilitated relationship building and the development of common language and perspectives around health and potential health impacts, it supported the iterative development of our knowledge translation efforts, and supported the conduct and evaluation of the Oyu Tolgoi HIA.

Although it is not possible at this point to measure the precise impact that the six years of policy advocacy and capacity building has had on changes in the coverage of health in EIA in practice, or in terms of furthering institutionalization of HIA practice more broadly, reflection on efforts to advance HIA in Mongolia over this period, and especially recent promising changes to the regulatory environment, suggest that projects such as those we describe here play an important role in creating awareness, strengthening buy-in, and building capacity at the level of policy and governance. It is clear that the intensive training activities provided an important forum for dialogue between key stakeholders to occur, and that this underpinned progress following the events. Whether these efforts will continue to lead to substantive changes to policy implementation and practice over time will become evident in the future when there is an opportunity to reflect on actual impacts and to detect any changes in the number and/or quality of EIAs and HIAs conducted.

It is useful to consider the six years of effort by our international partnership and a growing number of local stakeholders in a context of theories of policy change. As articulated by Shearer et al. [24], traditional models of policy change include the ‘3I’s’ of “institutions, interests, and ideas,” but ignore the importance of policy networks in either driving or constraining health policy change. In their case study analysis of three domains of policy change in Burkina Faso, Shearer et al. observed that while the “3I’s” are clearly important to create the conditions for policy change, networks are implicated in promulgating and disseminating interests and ideas. In a similar fashion, our policy intervention efforts focused initially on several key domains within the ‘3I’s’: bringing new ideas about HIA and EIA to the Ministries of Health, and Ministry of Environment, and other related institutions and applying a number of techniques – workshops, stakeholder consultations, and community engagement activities – to create constituencies within these institutions interested in transforming policy to include a more robust orientation to community health. Essential to these efforts were the effective establishment of stakeholder networks that crossed traditional sector boundaries. Especially in new democracies like Mongolia, where elections often foster large shifts in Ministry personnel, these cross-sectoral linkages provide a stable base of idea and policy advocates whose influence may transcend election cycles.

## Conclusions

It is clear that Mongolia’s EIA system will be important for the country to meet its goals for sustainable development. The system can also be used to enhance governance of health issues that are connected with the rapid development of Mongolia’s mining economy. However, as the experiences described in this paper show, harnessing this health potential is a formidable challenge with a myriad of moving parts. Integrating health into a given EIA system requires good intersectoral collaboration backed by a solid understanding of the culture and regulations that define that system. This takes both time as well as a willingness for staff from different Ministries, who often work in isolation, to work closely together. Participation of key stakeholders from the private sector, particularly those operating under IFC-promulgated performance standards that reinforce the prominent role of health in impact assessments, is also important, and may provide opportunities for developing public-private partnerships with the potential to protect the public’s health if HIA recommendations are implemented [9].

Our approach to effecting policy change illustrates two key points about networks and partnerships in global health. First, effective cross-disciplinary partnerships are needed to create the necessary inputs to policy change. In this case we were benefitted by a long history of collaboration between Canadian and Mongolian academic researchers and public health practitioners. With key supports provided by WHO and several Canadian funding sources, individual, time-limited projects each capitalized and built on the successes of previous efforts, facilitating the diffusion of important policy innovations. Secondly, these initial partnerships led to others, in particular a catalyzing of relationships between divisions within the Ministries of Health and Environment [20]. In this sense, our approach highlights a central tenet of the Health in All Policies movement: improvements to health are best achieved by incorporating health considerations into policymaking across sectors and policy domains [25]. Given the increasing demands placed on emerging economies to accept the terms of extractive industry development, ensuring that health concerns are and remain central to the policy agenda is a constant challenge. As we have shown here, a focus on aligning health impacts with regulated EIA processes, and providing opportunities for the dissemination of ideas that are supported by effective partnerships foster the catalyzing of key cross-sectoral relationships and institutional changes, offers a promising path forward.

We believe that there are four key lessons that can be drawn from our work that are applicable to other LMIC settings. First, in a context where officials change posts with each change of government (unfortunately common in many countries), it is important to focus on building up networks of expertise and strong partnerships that transcend ministerial silos. Over the six years that we describe here, several key health and environment staff changed positions or left civil service. These shifts cannot be predicted in advance, and those who leave civil service may only do so temporarily. A focus on creating a broad pool of expertise with relationships that cross many sectors – academic, civil society, private concerns, multiple government ministries – provides for a strong intellectual foundation and community of practice that will continue to support a health agenda within the EIA process. Secondly, creating a strong conceptual foundation for understanding the broad determinants of health is essential in order to push back at the reductionism that typically characterizes the impact assessment process. While we accept that social determinants of health, health equity or Health in All Policies approaches are aspirational and may not be suited to all contexts, some acceptance of core concepts is needed to move forward with a robust assessment of the health effects of development and the multi-sector responses that are inevitably required to address such effects [9]. Thirdly, as we found in Mongolia, building a strong and flexible partnership based on principles of equity and shared decision-making is essential. Rooted initially in a partnership struck by the Canadian Coalition for Global Health Research, our group grew to embrace many individuals, institutions and experts.^5^ This open partnership gave us the flexibility to respond to various opportunities and constraints. Finally, and in hindsight, it would have been impossible at the beginning to script a single, overarching intervention to bring health impact assessment into the Mongolia EIA system. Each project was built on previous ones, and supported by our flexible partnership, were able to respond to developments, legislative wins, and funding opportunities, bringing in different levels of technical expertise as needed [19]. This iterative approach, particularly in a context of integrated knowledge translation and policy advocacy, helped enhance the diffusion of a particularly important innovation to governance of the extractive sector.

## Abbreviations

CCGHR: Canadian Coalition for Global Health Research
CIRDI: Canadian International Resource Development Institute
EIA: Environmental impact assessment
GDP: Gross domestic product
HIA: Health impact assessment
HIA-LDP: Health impact assessment learning development programme
MOE: Ministry of Environment (now named the Ministry of Environment, Green Development and Tourism); see notes.
MOH: Ministry of Health (in Mongolia has had two other names over the course of this project – Ministry of Health and Sport; and Ministry of Health and Social Welfare); see notes.
SDGs: Sustainable Development Goals
UNFPA: United Nations Population Fund
UNICEF: United Nations Children’s Fund
UN-ILO: United Nations International Labour Organization
WHO: World Health Organization

## Footnotes

1 This partnership included researchers and policymakers from the Ministry of Health and Sport of Mongolia (MOH), the Ministry of Environment, Green Development and Tourism (MOE), the Ministry of Mining, Simon Fraser University (Canada), the University of Waterloo (Canada), Queens University (Canada), the Canadian Coalition for Global Health Research (CCGHR), and local Mongolian public health NGOs, with technical support from WHO and funding support from Canada.

2 The names of Mongolia Ministries changed over the time period discussed here. For ease of exposition and to avoid confusion we refer simply to the Ministry of Health (which became at one point the Ministry of Health and Social Welfare, and more recently is named the Ministry of Health and Sport), and to the Ministry of Environment (which later became the Ministry of Environment, Green Development and Tourism).

3 Oyu Tolgoi (“turquoise hill”) is a major copper mine in the southern Gobi desert region. It is operated as a joint venture between Turquoise Hill Resources (a subsidiary of the global mining company Rio Tinto) and the Government of Mongolia. The mine began operation in 2010. The Health Impact Assessment was conducted as part of the mine’s commitment to International Finance Corporation performance standards, in particular to ensure “Community Health, Safety, and Security.” Energy Resources, LLC is a Mongolia owned and operated coal mining company that operates the Ukhaa Khudag mine in the Tavan Tolgoi (“five hills”) region of the southern Gobi desert. It is approximately 120 km to the north of Oyu Tolgoi. Operations began in 2009. Energy Resources conducted environmental, social, and economic impact assessments as required by The European Bank for Reconstruction and Development, and maintains an active community-engagement program.

4 In this case, the company was following International Finance Corporation (IFC) performance standards. These standards, while voluntary, have been adopted by most major multinational financial institutions (e.g., “Equator Principles” banks). Projects requiring financing capital are typically required to meet these standards, which often require completion of a suite of impact assessments, including health impact assessments, as well as to establish avenues for community participation and grievance resolution [9].

5 The Canadian Coalition for Global Health Research has given considerable attention to developing principles for ethical partnerships. With funding from the International Development Research Centre of Canada, and conducted in partnership with several international organizations, it has developed a useful partnership assessment tool (PAT). The PAT is freely available and accessible on the CCGHR website: http://www.ccghr.ca/resources/partnerships-and-networking/partnership-assessment-tool/
